# IMU-Based Classification of Locomotion Modes, Transitions, and Gait Phases with Convolutional Recurrent Neural Networks

**DOI:** 10.3390/s22228871

**Published:** 2022-11-16

**Authors:** Daniel Marcos Mazon, Marc Groefsema, Lambert R. B. Schomaker, Raffaella Carloni

**Affiliations:** Bernoulli Institute for Mathematics, Computer Science and Artificial Intelligence, Faculty of Science and Engineering, University of Groningen, Nijenborgh 9, 9747 AG Groningen, The Netherlands

**Keywords:** lower-limb prosthetic, deep neural networks, motion classification

## Abstract

This paper focuses on the classification of seven locomotion modes (sitting, standing, level ground walking, ramp ascent and descent, stair ascent and descent), the transitions among these modes, and the gait phases within each mode, by only using data in the frequency domain from one or two inertial measurement units. Different deep neural network configurations are investigated and compared by combining convolutional and recurrent layers. The results show that a system composed of a convolutional neural network followed by a long short-term memory network is able to classify with a mean F1-score of 0.89 and 0.91 for ten healthy subjects, and of 0.92 and 0.95 for one osseointegrated transfemoral amputee subject (excluding the gait phases because they are not labeled in the data-set), using one and two inertial measurement units, respectively, with a 5-fold cross-validation. The promising results obtained in this study pave the way for using deep learning for the control of transfemoral prostheses with a minimum number of inertial measurement units.

## 1. Introduction

Research on micro-controlled lower limb prostheses focuses on providing them with the ability of accurately understanding the user’s intention to achieve an intuitive use and, ultimately, to improve the user’s quality of life. Moreover, to reduce any discomfort in using the prosthesis, its response to a specific intention should occur within 300 ms [1].

Table 1 summarizes the main contributions in the literature for the classification and the prediction of locomotion modes, transitions, and gait phases, and it indicates the used methods (either machine learning or deep neural networks), their accuracy or error, the used sensors and their placement on the subject’s body, and whether testing is done on healthy or impaired subjects.

This paper focuses on the combined classification of locomotion modes, the transitions among these modes, and the gait phases within each mode for healthy and transfemoral amputee subjects, by relying on a minimum number of inertial measurement units (IMUs).

This work builds upon our previous studies on the classification of locomotion modes for ten healthy subjects [9], and on the prediction of locomotion modes and transitions for one transfemoral subject [10]. To jointly classify a high number of classes for both healthy and transfemoral amputee subjects without the need for engineering the features, we propose a novel multi-level architecture composed of multiple deep neural networks. Specifically, we investigate different architectures in which convolutional neural networks (CNN) are combined with long short-term memory (LSTM) layers and gated recurrent unit (GRU) layers. As inputs to the networks, the spectrograms of IMU data, either from one IMU (placed on the upper leg) or two IMUs (placed on both the upper and lower leg), are used. The system is trained to classify seven locomotion modes (sitting, standing, level ground walking, ramp ascent and descent, stair ascent and descent), the transitions among them (twelve transitions in the ENABL3S public data-set [24] for ten able-bodied subjects, and nineteen transitions in the MyLeg data-set for one osseointegrated transfemoral amputee), and the twenty-seven gait phases within each mode (only for the ENABL3S data-set because the MyLeg data-set does not have gait phase labels). This study shows that a multi-level architecture made of CNN-LSTM neural networks can classify locomotion modes, transitions, and gait phases for ten healthy subjects with a mean F1-score of 0.89 ± 0.006 using one IMU and 0.91 ± 0.01 using two IMUs, and that the same CNN-LSTM multi-level architecture can classify locomotion modes and transitions for one transfemoral amputee with a mean F1-score of 0.92 ± 0.01 using one IMU and 0.95 ± 0.01 using two IMUs. To summarize, the main contributions of this paper are:

- Design a novel multi-level architecture made of different deep neural networks for the classification of a high number of classes (seven locomotion modes, twelve or nineteen transitions, and twenty-seven gait phases). In the current literature, multi-level architectures have been proposed in [4,22], where feature engineering methods (SVM and QDA, respectively) are used for the prediction and/or classification of locomotion activities and phases. However, besides not using feature learning methods, the considered number of classes is lower than in this study (i.e., fourteen and eighteen, respectively).
- Use data only from either one or two IMUs and, specifically, their frequency information as input to the neural network architectures. In the current literature, features in the frequency domain from IMU data have been used for classification in combination with other sensors’ data in [5], and with the same sensors setting in our previous work [10].
- Validate the results with two different data-sets, one with ten healthy subjects and the other with one osseointegrated transfemoral amputee, the former being able to classify locomotion modes, transitions, and gait phases and the latter only locomotion modes and transitions. Even if deep neural networks have been previously used to classify locomotion modes by using IMU data, this study extends to a high number of classes and considers data-sets of both healthy and transfemoral subjects.
- Obtain, with a CNN-LSTM architecture, a top F1-score of 0.91 ± 0.01 with two IMUs for the healthy subjects and 0.95 ± 0.01 with two IMUs for the amputee subject, with a 5-fold cross-validation. While machine learning techniques still outperform deep neural networks [22,23], this study shows the potential of feature learning techniques for the classification and, possibly, prediction of a high number of locomotion activities and phases, while, at the same time, relying on a limited number of sensors.

The remainder of this paper is organized as follows: Section 2 presents the data-sets used in this study and their pre-processing. Section 3 explains the multi-level architecture and the different deep neural networks that have been designed and tested. Section 4 presents and discusses the obtained results. Concluding remarks are drawn in Section 5.

## 2. Materials

This section presents the two data-sets and the pre-processing to extract the sequences used as inputs to the deep neural networks.

### 2.1. Data-Set

#### 2.1.1. ENABL3S Data-Set

The publicly available ENABL3S (Encyclopedia of Able-bodied Bilateral Lower Limb Locomotor Signals) data-set [24] contains IMU data (accelerometer and gyroscope data) from ten able-bodied subjects. The data were gathered from seven males and three females with an average age of 25.5 ± 2 years, a height of 174 ± 12 cm, and a weight of 70 ± 14 kg. From this data-set, only data from the two IMUs located on the upper and lower leg are used. The data are sampled at 500 Hz by means of the MPU-9250 (InvenSense, San Jose, CA, USA), i.e., there are new data every 2 ms. The available locomotion modes are: sitting (S), standing (ST), level ground walking (W), ramp ascent (RA) and descent (RD), stair ascent (SA) and descent (SD). The ramps have slopes of 10${}^{\circ }$, and the stairs consist of four steps.

**Figure 1:**
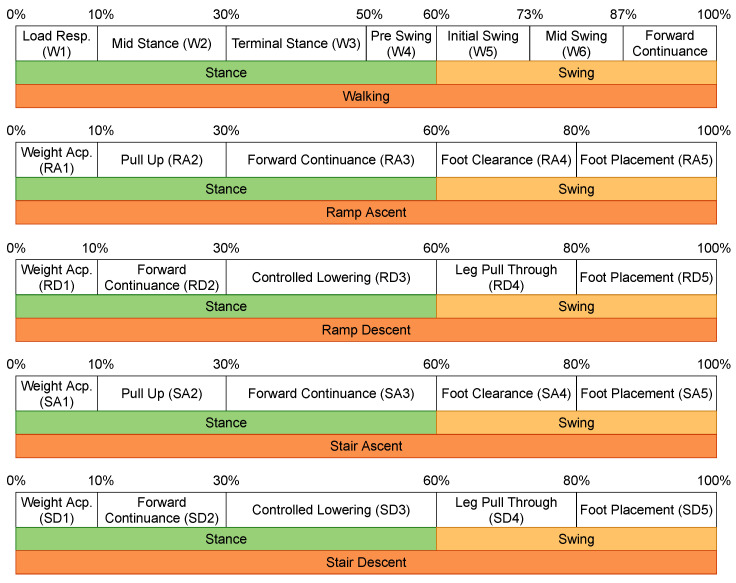
Locomotion modes and corresponding gait phases.

The original data-set only provides the labels of the locomotion modes, but it also contains information that helps with obtaining the transitions between two locomotion modes, as well as the gait phases. To extract the transitions, a 500 ms window centered at a transition point (the time step in between two subsequent locomotion modes) is created [10], and the data inside are labeled according to the transition, e.g., the transition from walking to sitting is labeled as W–S. The gait phases are obtained by means of the toe-off and heel-strike events, and are linked to a specific locomotion mode, as shown in Figure 1 (retrieved from [25,26,27]). Note that sitting and standing do not have any gait phase information since they are static modes.

#### 2.1.2. MyLeg Data-Set

This data-set was collected at the Roessingh Research and Development Center (Enschede, The Netherlands) on one osseointegrated transfemoral amputee subject (male, 75 years old, 84.1 kg, 186.6 cm, left-sided amputation since 45 years, osseointegration since 4 years, functional level K3), using a 3R80 Ottobock prosthetic knee (www.ottobockus.com, accessed on 13 November 2022) and a Variflex Össur prosthetic ankle (www.ossur.com, accessed on 13 November 2022).

The data were collected from the subject by using wearable electromyographic sensors and eight IMUs, as part of the Xsens MVN Link motion capture system (Xsens Technologies B.V., Enschede, The Netherlands, www.xsens.com, accessed on 13 November 2022). In this study, data from two IMUs (one on the left upper leg and one on the left lower leg) are used. The IMU data are sampled at a frequency of 1000 Hz, which means there are new data every 1 ms. The available locomotion modes are: sitting (S), standing (ST), level ground walking (W), ramp ascent (RA) and descent (RD), stair ascent (SA) and descent (SD). The ramps have a slope of 10${}^{\circ }$ for three meters, and continue on with a slope of 15${}^{\circ }$.

The original data-set only provides the labels of the locomotion modes. To extract the transitions, the same procedure as for the ENABL3S data-set has been used but with a 500 ms window centered at the transition point.

### 2.2. Data Processing

#### 2.2.1. Sequence Extraction

The inputs to the deep neural networks are sequences, which are formed by a portion of sequential data from either one of the used data-sets. The data contain information from the IMU, i.e., in the case of one IMU, the data are from one triaxial accelerometer and one triaxial gyroscope for a total of six features, while, in the case of two IMUs, the data are from two triaxial accelerometers and two triaxial gyroscopes for a total of 12 features. The size for the sequences is 1.3 s (empirically found), with a sliding window of 50 ms (also empirically found). This means that, for the ENABL3S data-set, a sequence contains 650 data while, for the MyLeg data-set, a sequence contains 1300 data.

**Figure 2:**
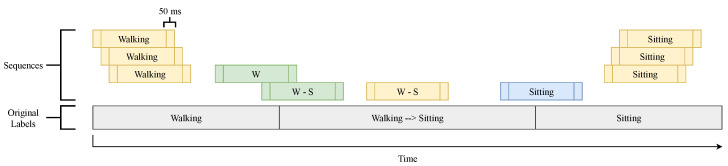
Example of sequence extraction. The original data-set is represented in grey with its original labels. The sequences (with a size of 1.3 s and a sliding window of 50 ms) are represented in yellow/green/blue and are relabeled as explained in Section 2.2.1.

When extracting sequences from the original labeled data-sets, three different scenarios can occur: (i) the extracted sequence falls completely in one locomotion mode or transition and, therefore, it gets labeled as such; (ii) the sequence falls in between a locomotion mode and a transition and, therefore, it receives the label of the majority of the data (≥50%) contained in said sequence; (iii) the sequence is in between a transition and a locomotion mode and, therefore, it gets labeled as the locomotion mode. Figure 2 depicts an example of multiple sequence extractions. The yellow sequences represent case (i), in which the extracted sequence falls entirely into one locomotion mode or transition and thus it is labeled as such. The green sequences represent case (ii), where the top sequence contains more data from the walking locomotion mode and is labeled as walking, while the bottom sequence has more data from the transition Walking to Sitting and so it is labeled as W–S. The blue sequence represents case (iii), where the sequence starts in a transition and finishes in a locomotion mode and is labeled as the locomotion mode. This last case is not labeled as a transition since the main interest resides at the beginning of the transition rather than at the end. Thus, by labelling it as the next locomotion mode, it will not interfere in the transition classification. Table 2, Table 3 and Table 4 show the number of sequences for the locomotion modes, transitions, and gait phases in the two data-sets.

#### 2.2.2. Frequency-Domain Encoding

**Figure 3:**
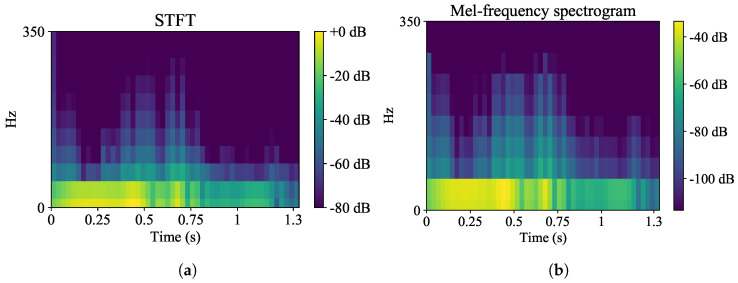
Initial spectrogram (**a**) and mel spectrogram (**b**) from one of the IMUs signals. The higher frequencies are attenuated after the mel scaling.

The IMUs raw data are encoded in the frequency-domain by using a spectrogram [5,10], since it allows for taking advantage of the periodic nature of the human movement. Firstly, the short-time Fourier-transform (STFT) is computed to obtain the frequency–domain information from the time-series data. Then, after squaring the output signal, the spectrogram is modified by means of a nonlinear mel scale [5], which amplifies the lower frequencies where most of the human movement information can be found (below 3.5 Hz [28]), as shown in Figure 3. The mel scaling can be computed as $2595\cdot \log_{10}\left(1+f/700\right)$, where 2595 is a constant value ensuring that 1000 Hz corresponds to 1000 mel, and 700 is the corner frequency at which the scales changes from linear to logarithmic [29]. The signal is then converted into dB and normalized in the [0, 1] range so it can be processed by the neural networks. For the calculation of the STFT, a Hann window of size 20 with an offset of 13 is used. When using the mel scale, the Hz scale is partitioned in 10 bins in order for some channels in the mel spectrogram to not return an empty response. For implementation, the Python package LibROSA [30] is used.

#### 2.2.3. Data Partitioning

The data-set has been divided as follows: 80% of the data was used for training, and 20% was used for validation. Within training, 20% of the data was used for testing.

## 3. Methods

This section presents the overall system architecture, as well as the different neural networks, implemented either with a CNN, CNN-GRU, or CNN-LSTM, as inspired by our previous work [9,10]. In the end, an overview of the experimental setting is given.

### 3.1. System Architecture

**Figure 4:**
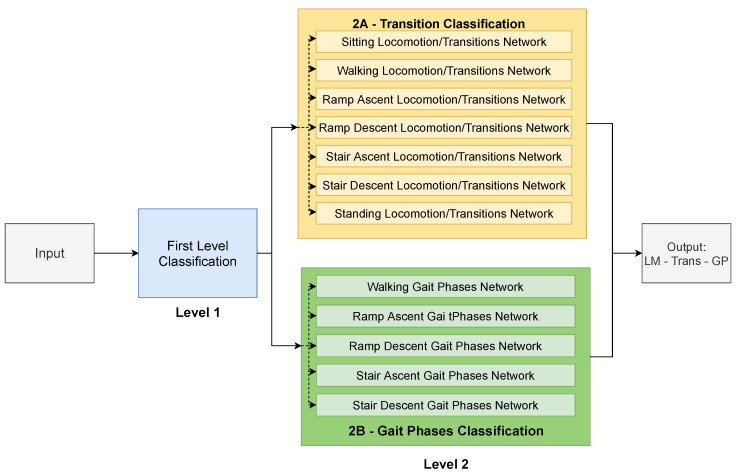
Proposed multi-level architecture for the classification of the locomotion modes, transitions, and gait phases. Level 1 performs a first general classification of locomotion modes, while level 2 distinguishes between locomotion modes and transitions as well as gait phases.

This study proposes a multi-level architecture as depicted in Figure 4. The input, i.e., the spectrogram of the input sequence, goes into a first level classification (blue box in Figure 4), which classifies to which locomotion mode the input sequence belongs. It is composed of one single neural network and, here, locomotion modes and transitions sequences are treated the same. This means that, for example, sequences labeled as the walking locomotion mode, and sequences labeled as transitions that start with walking, are both labeled as walking. This level works as a pre-classification step for the next level. The second level is made of two parts: one is in charge of classifying locomotion modes and transitions (level 2A), and the other one is in charge of classifying gait phases (level 2B). Level 2A is composed of seven different neural networks, each one in charge of a different locomotion mode. Depending on the result of level 1, the input sequence goes into one or another. While locomotion modes and transitions are treated equally in level 1, they get classified as either one of the locomotion modes or one of the transitions in level 2A. For example, a walking to standing transition that was classified as walking by level 1 would be classified as W–ST in level 2A. In the case of having a walking sequence classified as walking by level 1, it would be classified as W–W by level 2A, thus making the distinction from locomotion modes and transitions. Level 2B is composed of five different networks, one for each locomotion mode that has gait phases (i.e., sitting and standing are excluded). The input sequence goes into one network or another depending on the result of level 1, independently of being a locomotion mode or a transition. Note that, for the MyLeg data-set, level 2B is ignored since there are no gait phases to be classified. It should be also noted that level 2A and level 2B are independent from each other, thus they could be implemented both sequentially and in parallel. In this study, it was decided to implement them in parallel.

**Figure 5:**
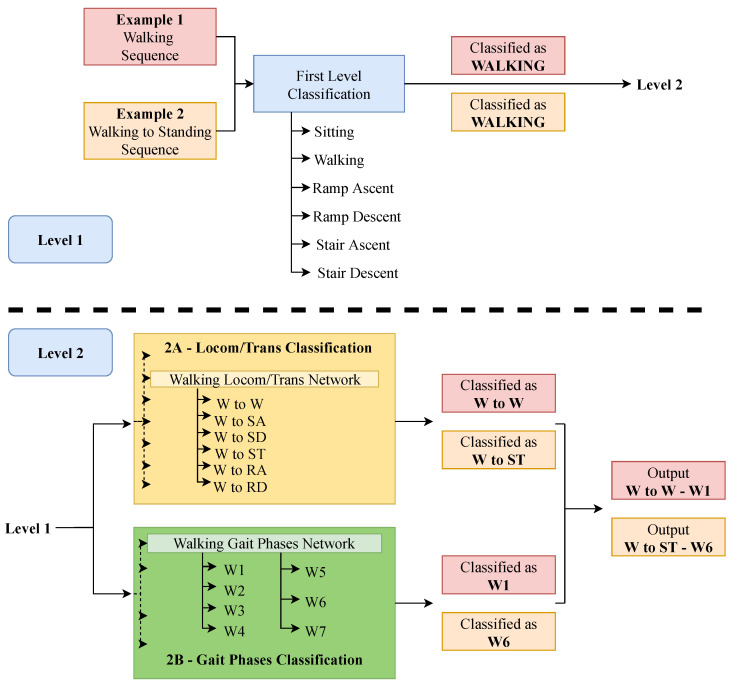
Example of classification of a given sequence.

Figure 5 shows an example of how an input sequence is processed through the system architecture. Two input sequences are shown: one sequence corresponds to the walking locomotion mode with gait phase W1 (red sequence), and another sequence corresponds to a walking to standing transition with gait phase W6 (orange sequence). In the first level, both of them are classified as W. In level 2A, the sequences go into the “Walking Locomotion/Transitions Network”, being classified as W to W (red sequence) and W to ST (orange sequence). Something similar happens in level 2B, where both sequences enter the “Walking Gait Phases Network” and are classified as W1 (red sequence) and W6 (orange sequence). The final results are “W to W–W1” for the red sequence and “W to ST–W6” for the orange sequence.

### 3.2. Convolutional Neural Network

**Figure 6:**
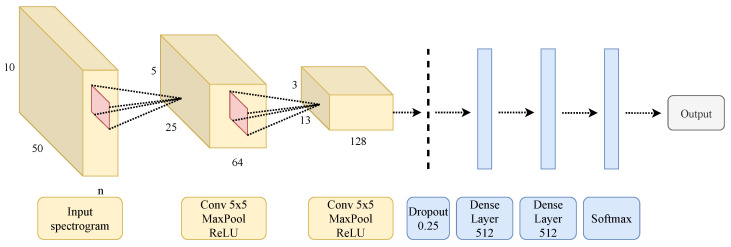
CNN architecture.

Figure 6 shows the architecture for the convolutional neural network. The input to the network is the mel-spectrogram image, which has a size of 10 × 50. The first two layers are convolutional layers with 5 × 5 kernel size, and the number of filters equals 64 and 128, respectively. They use a rectified linear unit and a max-pooling of size 2 × 2. Finally, there is a dropout layer with a value of 0.25, two dense layers of sizes 512 and 256, respectively, and a softmax layer whose size depends on the output of the network. For example, the network classifying walking gait phases in level 2B has a size of 7, while the network classifying ramp descent gait phases has a size of 5.

### 3.3. Convolutional Recurrent Neural Network

**Figure 7:**
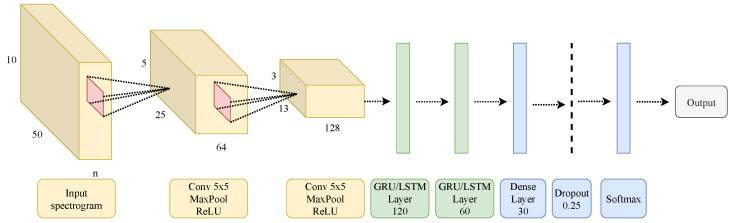
CNN-(LSTM/GRU) architecture.

Figure 7 shows the architecture for the convolutional recurrent neural network (CNN-GRU or CNN-LSTM) [9]. The input to the network is the mel-spectrogram image, which has a size of 10 × 50. The first two layers are convolutional layers with 5 × 5 kernel size, and the number of filters equals 64 and 128, respectively. The recurrent layers follow, which are either two GRU layers or two LSTM layers with 120 and 60 units, respectively. Finally, there is a dense layer with a size of 30, a dropout layer with a value of 0.25, and a softmax layer whose size depends on the output of the network.

### 3.4. Evaluation: Performance Metric

As it can be seen in Table 2, Table 3 and Table 4, there is an imbalance among the number of sequences in the different classes. Because of this, the F1-score is used to measure the performance of the deep neural networks. It can be calculated as F1=2·(precision·recall)/(precision+recall), where precision=tp/(tp+fp) and recall=tp/(tp+fn), with tp being the number of true positive classifications, fp the number of false positives, and fn the number of false negatives. For a classification to be considered correct, both the locomotion mode/transition and the gait phase must be correct. This means that it is an end-to-end measurement of the performance of the system (the F1-score for level 2A and level 2B are also not independent), with the only drawback being that, if level 1 misclassifies a sequence, the error will be propagated to the rest of the network.

### 3.5. Hyperparameters

This subsection describes the main hyperparameters that were taken into account during the training process. The values for these hyperparameters are used as a baseline, since later on a grid search is conducted on each individual network to optimize its performance. The training is done on one computer with a GeForce RTX 2080 SUPER, AMD Ryzen 7 3700X 8-Core Processor, and 8 GB RAM.

#### 3.5.1. Learning Rate

The learning rate is set at 0.0001. A high value for the learning rate could cause the network to never converge while a very low learning rate could cause the network to get stuck in a local minima, thus the chosen value.

#### 3.5.2. Optimizer

As optimizers, the Adaptive Moment Estimation (Adam) optimizer is used to optimize the gradient descent while training the network [31]. Adam computes individual adaptive learning rates for different parameters. The Root Mean Square Propagation (RMSProp) is considered as well as an optimizer during the network grid search optimization.

#### 3.5.3. Loss Function

The categorical cross-entropy is used as loss function.

#### 3.5.4. Class Weighting

The class weight function provided by the sklearn.utils module of the scikit-learn Python library is used to deal with the class imbalance in the data used for training [32]. This way, without the need for creating augmented data or varying the number of data in the data-set, the network penalizes the classification errors for the underrepresented classes more (i.e., the transitions).

#### 3.5.5. Epochs and Early Stopping

The data are presented 400 times to the networks during training to ensure that the data are used enough and are not under-fitting. If necessary, early stopping is used to stop the training if there has not been a sufficient improvement on the validation loss for 10 epochs. The minimum difference to consider an improvement in the validation loss is 0.001.

### 3.6. Experimental Steps

In this study, there are a number of steps taken from the optimization of the neural networks to obtain the final results for each data-set, i.e.,:

- Train each deep neural network architecture both for one and two IMUs;
- Use the results from the previous step to find the best performing architecture by using a paired *t*-test;
- Optimize the best performing network architectures with the grid search;
- Train and test on the ENABL3S data-set on a subject dependent basis, meaning that 80% of the data from the testing subject are included in the training process;
- Train and test on the ENABL3S data-set on a subject independent basis, meaning that the data from the testing subject are completely excluded from the training process;
- Train and test on the MyLeg data-set;
- Test the effect of training with healthy subjects data (ENABL3S) and testing on the amputee subject (MyLeg);
- Evaluate the classification time of the system.

## 4. Results

This section presents the results obtained in the different experiments listed above. The results are reported separately for each experimental step and by distinguishing between the use of data from one or two IMUs.

### 4.1. Individual Network Optimization

As shown in Figure 4, there are many neural networks in the final system, each one in charge of classifying a certain subset of classes. In this step, all the different network architectures are taken into consideration for each one of the classification problems, both for one or two IMUs. The networks are trained and tested on each subject of the ENABL3S data-set, and the final F1-score is the result of averaging individual F1-scores.

**Figure 8:**
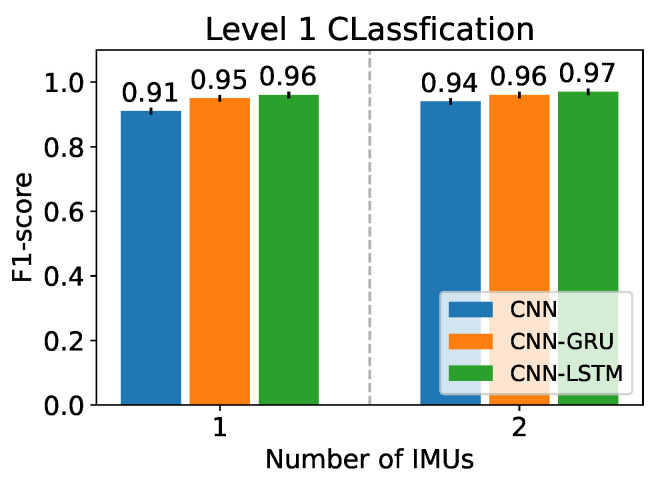
F1-score of the different network architectures for one and two IMUs for the classification task of level 1.

**Figure 9:**
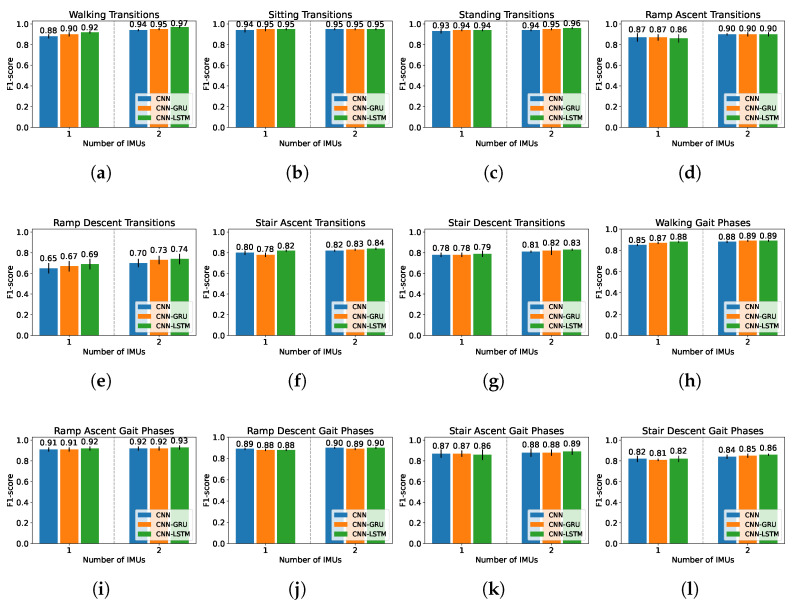
F1-score of the different network architectures for one and two IMUs for the classification tasks of level 2. (**a**) Walking Transitions; (**b**) Sitting transitions; (**c**) Standing transitions; (**d**) Ramp ascent transitions; (**e**) Ramp descent transitions; (**f**) Stair ascent transitions; (**g**) Stair descent transitions; (**h**) Walking gait phases; (**i**) Ramp ascent gait phases; (**j**) Ramp descent gait phases; (**k**) Stair ascent gait phases; (**l**) Stair descent gait phases.

Figure 8 reports the initial F1-scores of the networks in level 1. The CNN-LSTM performs the best with F1-scores of 0.96±0.01 with one IMU, and 0.97±0.01 with two IMUs. Figure 9 reports the initial F1-scores of the networks in levels 2A and 2B. The CNN-LSTM performs the best with an F1-scores ranging from 0.69±0.06 to 0.95±0.03 with one IMU, and from 0.74±0.05 to 0.95±0.01 with two IMUs. Additionally, according to a paired *t*-test, there are significant differences between the CNN-LSTM and the other architectures (CNN and CNN-GRU) with *p*-values of $6.20\times 10^{-5}$ and $3.47\times 10^{-5}$ in level 1 (Figure 8), and with *p*-values of 0.001 and 0.021 in level 2 (Figure 9). Therefore, the CNN-LSTM architecture is used for the subsequent steps, i.e., for the hyperparameter optimization and the final experiments.

A grid search with a 5-fold cross validation is used for the hyperparameter optimization. Table 5 reports the best performing set of hyperparameters values and thus the configuration each neural network has for the remainder of this study.

### 4.2. ENABL3S Subject Dependent

**Figure 10:**
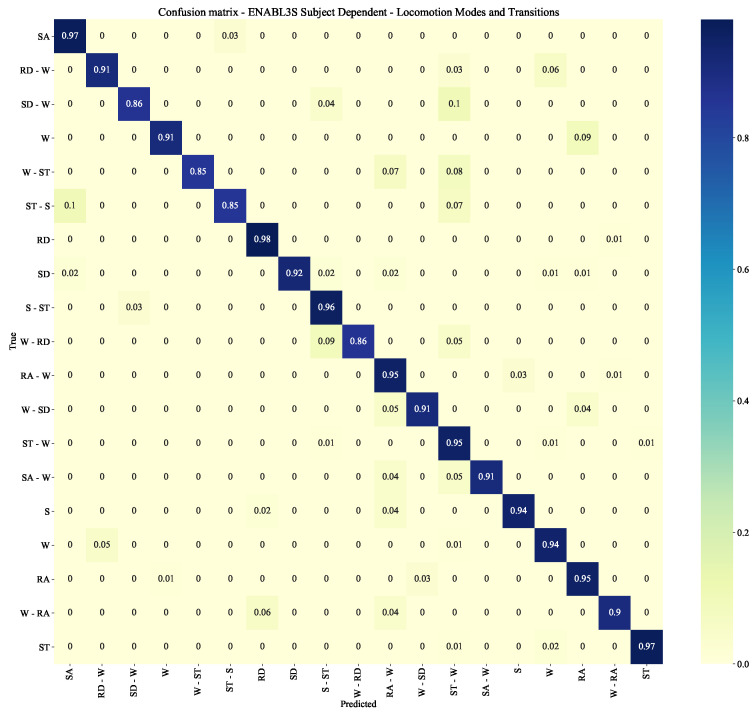
Confusion matrix for the ENABL3S subject dependent scenario with an CNN-LSTM for the locomotion modes and transitions’ classification.

**Figure 11:**
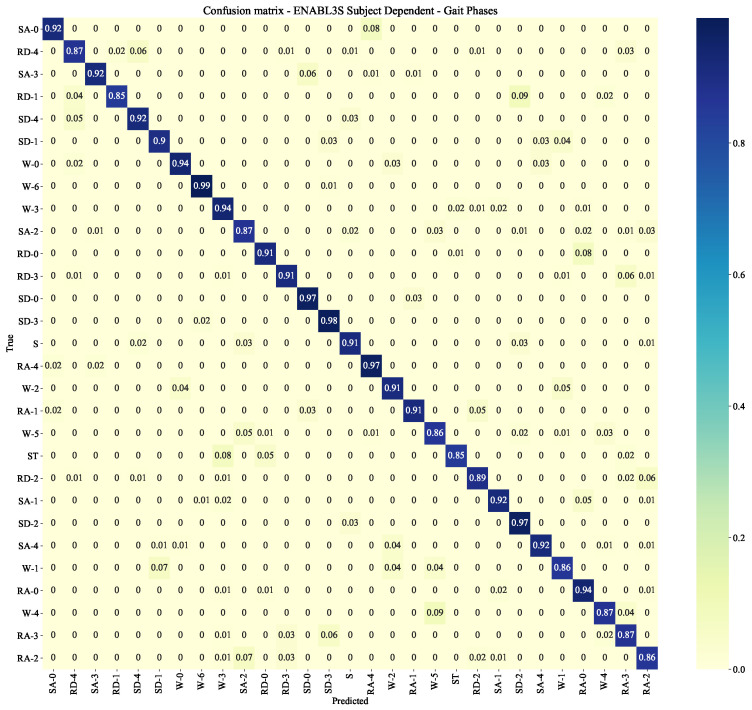
Confusion matrix for the ENABL3S subject dependent scenario on the CNN-LSTM configuration for the gait phases classification.

This experiment consists of training the system on each healthy subject of the ENABL3S data-set, independently of each other, and test it on itself. The objective is to evaluate the base performance of the networks on a personal subject, where all data used both for training (80% of the data) and testing (20% of the data) belong to the same user. The F1-scores are obtained after averaging the individual F1-score of the ten subjects. The system achieves an F1-score of 0.89 ± 0.01 and of 0.91 ± 0.01 with one or two IMUs, respectively. Additionally, it obtains an F1-score of 0.93 ± 0.01 in the locomotion modes and transition classification (level 2A), and an F1-score of 0.95 ± 0.01 in the gait phases classification (level 2B). Figure 10 and Figure 11 report the corresponding confusion matrices, where it can be noticed that some classes obtain an F1-score of ∼0.85. These scores mainly belong to transitions, either transitions not being classified correctly or locomotion modes that are mistakenly classified as transitions. The reason for this could be inferred to transition classes being under-represented.

### 4.3. ENABL3S Subject Independent

This experiment tests the generalization capabilities of the system by testing on a novel subject. From the ENABL3S data-set, nine subjects are used for training, and the remaining one is used for testing. After that, the network is retrained with 80% of the data from the missing subject to see how much the network is able to improve. This process is repeated until all subjects have been used once for testing. Before retraining with the missing data, the system achieves an F1-score of 0.50 ± 0.03 and of 0.61 ± 0.05 with one or two IMUs, respectively. After retraining, the system achieves an F1-score of 0.89±0.02 and of 0.91±0.02 with one or two IMUs, respectively. This lack of generalization might be caused by the fact that different subjects move in different ways. Even though a locomotion mode across multiple subjects is essentially very similar, differences in aspects such as the speed or the range of movement of the limbs might produce a reduction in the overall performance of the system’s classification.

### 4.4. MyLeg Subject Dependent

**Figure 12:**
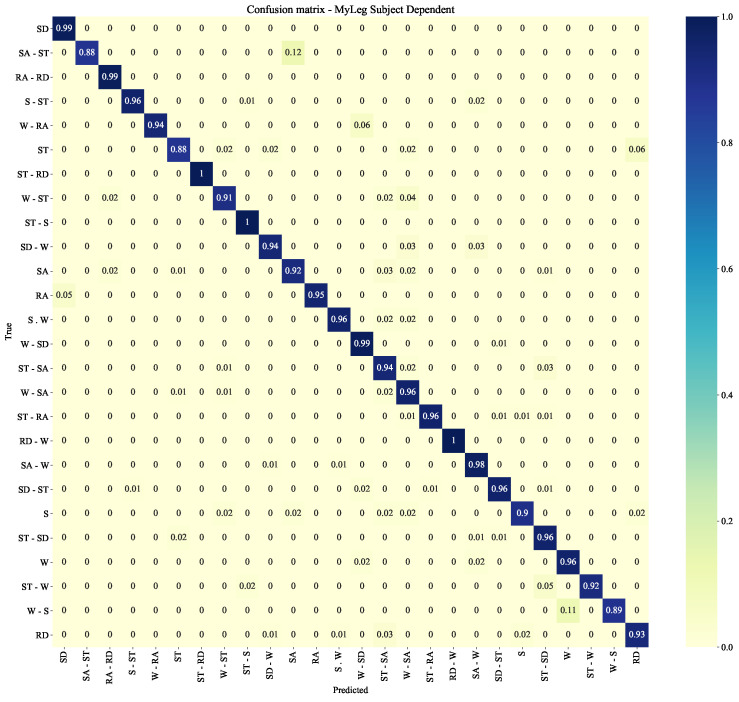
Confusion matrix for a MyLeg subject dependent scenario on the CNN-LSTM configuration.

In this experiment, the system is tested on the osseointegrated transfemoral amputee of the MyLeg data-set. Note that, in this case, there is no gait phase classification. The system achieves an F1-score of 0.92 ± 0.01 and of 0.95 ± 0.01 with one or two IMUs, respectively. Figure 12 reports the corresponding confusion matrix, where it can be noticed that some classes obtain an F1-score of ∼0.85, which could be inferred to the class distribution. The main difference with respect to the ENABL3S data-set is that, this time, there is no classification coming from level 2B as there are no gait phases involved, which makes the multi-level architecture be less influential on the overall F1-score.

### 4.5. MyLeg and ENABL3S Subject Independent

This experiment tests the performance of mixing healthy with amputee data. Initially, the system is trained with data from either one or ten healthy subjects of the ENABL3S data-set and, then, tested on the amputee data of the MyLeg data-set. After that, the system is retrained with the amputee data and tested again to see if there is any improvement. Finally, the system is tested also on the healthy data to check the effect of retraining the networks with amputee data.

As summarized in Table 6, when using only one ENABL3S subject, the initial peak F1-scores are 0.24 ± 0.06 and 0.21 ± 0.04 with one and two IMUs, respectively. After retraining with the amputee data, they increase to 0.87 ± 0.01 and 0.95 ± 0.01, respectively. Testing on the healthy subject after retraining the system with the amputee data produces low performance, i.e., F1-scores of 0.33±0.02 and 0.37±0.03 with one and two IMUs, respectively.

As summarized in Table 7, when using ten ENABL3S subjects, the initial peak F1-scores are 0.29 ± 0.01 and 0.22 ± 0.02 with one and two IMUs, respectively. After retraining with the amputee data, they increase to 0.84 ± 0.02 and 0.95 ± 0.01, respectively. Testing on ten healthy subjects after retraining the system with the amputee data produces low performance, i.e., F1-scores of 0.24±0.03 and 0.34±0.01 with one and two IMUs, respectively.

From the tables, it can be concluded that testing the amputee data on a system trained with healthy data does not produce desirable results. This result can be explained by observing that healthy and amputee movements are not comparable given the differences in locomotion abilities between these two groups. After retraining with the amputee data, the F1-scores improve but not significantly when compared to the F1-score obtained in the subject dependent experiment with the MyLeg data-set. This also implies that there is no effect in pre-training the networks on a different number of subjects since the results will most likely be the same.

### 4.6. Running Time

Table 8 shows the average running time for the classification of one sequence, averaged over 1000 sequences, each one containing six or twelve spectrograms (one or two IMUs, respectively) extracted from 1.3 s of data. In both cases, the time necessary to perform the classification of one sequence is below 50 ms, which is the sliding window time that is set to obtain a new sequence, and it is also below 300 ms, which was the maximum time allowed not to cause any discomfort to the prosthesis’ user. It is worth noting that there is no clear difference between classifying data from one or two IMUs. The only difference is in computation time to obtain the spectrograms, which are computed with twice the signals. This overall computation time is calculated on a desktop computer, whose computational power is comparable with processors that can be placed on prosthetic leg prototypes.

### 4.7. Comparison to the State-of-the-Art

In this subsection, the results obtained in this study are compared to the literature, as summarized in Table 1.

#### 4.7.1. Locomotion Modes, Transitions, and Gait Phases

The proposed multi-level CNN-LSTM neural network can classify seven locomotion modes, the transitions among them (twelve transitions in the ENABL3S data-set and nineteen transitions in the MyLeg data-set), and the twenty-seven gait phases within each mode (only for the ENABL3S data-set). Previous research has been devoted to the disjoint classification/prediction of locomotion modes and/or transitions or of gait phases. Compared to [22,23] (where locomotion modes, transitions, and gait phases are jointly classified), this study makes use of only one or two IMUs (no force sensors nor pressure insoles are used) and considers a higher number of classes (especially for the gait phases).

With respect to the results obtained in [4], this study achieves comparable results while, however, using less IMUs (one or two instead of three), including twenty-seven gait phases in the classification, and extending the results to transfemoral amputees. With respect to the results in [5], this study achieves comparable results while, however, using six sensors less, including also gait phases in the classification, and extending the results to transfemoral amputees. With respect to the results in [9], this study achieves comparable results while, however, considering locomotion modes, transitions and gait phases instead of only seven locomotion modes, and extending the results to transfemoral amputees.

#### 4.7.2. Multi-Level Architectures

The proposed multi-level architecture differs from others in the literature. Specifically, in [4], a machine learning method (based on SVM) is used to extract the features from data in the time domain of three IMUs. Herein, the multi-level architecture distinguishes between steady-state and transitions in the first level and, in the second level, between five locomotion modes and nine transitions. In [22], a machine learning method (based on QDA) is used to extract the features from data in the time domain of 2 IMUs and a load cell. Herein, the multi-level architecture distinguishes between ambulation and standing in the first level, in the second level between stance and swing depending on the load cell and, in the third level, between five locomotion modes and ten transitions.

#### 4.7.3. Data-Sets

The proposed multi-level architecture builds upon our previous work in which the ENABL3S [9] and the MyLeg [10] data-set were used, and extends them to include the combined classification of locomotion modes, transitions, and gait phases. The study in [5] on the ENABL3S data-set outperforms our work, however excluding the gait phases.

#### 4.7.4. Generalization from Healthy to Amputee Subjects

This study has investigated the generalization capabilities among healthy and amputee subjects, showing that there is a poor generalization given the differences in locomotion gaits among these two groups, with little to no improvement when retraining the neural network with amputee data after initially training it with healthy data, when compared to training the neural network only with the amputee data. The improvements of the robustness of the proposed multi-level architecture for the generalization from healthy to amputee subjects are left for future work.

### 4.8. Limitations and Future Outlook

#### 4.8.1. Transition Extraction and Data-Set Structure

As reflected in Figure 10 and Figure 12, the transition classification achieves an average F1-score of ∼0.90. This might be due to the way in which transitions are extracted, which makes some transition sequences contain information about previous or subsequent locomotion modes. A more comprehensive study of the transition sequence extraction, together with the obtainment of transition information directly from the subjects, could help improve the final performance of the whole system as well by reducing the under-representation of transition data.

#### 4.8.2. Implementation

For the user to not feel any kind of discomfort, predictions must be made approximately within 300 ms. With the approach proposed in this study, one sequence, which is extracted from 1.3 s of data, is processed in ∼40 ms (about 4 ms to process the sequence and 37 ms to classify the sequence). This is not only inside 300 ms but also inside the 50 ms sliding window by which a new sequence is ready to be processed.

It must be taken into account that, for the experiments in this study, all necessary neural networks were loaded into memory at once since there was no need to optimize for memory efficiency. On a real prosthesis, this might not be possible, and neural networks need to be loaded every time they are needed and discarded right after, which might increase the amount of time necessary to process one sequence. On the other hand, in the experiments, every classification was run sequentially (level 1, then level 2A, then level 2B), so the running time can be improved if parallelism approaches are taken into consideration when designing the whole system.

#### 4.8.3. Clinical Requirements

Future research should focus on the implementation and evaluation of the proposed method on osseointegrated amputees in clinical trials. This study can be the starting point since the overall F1-score for the amputee subject reaches 0.95, with individual classes mostly above 0.9, as shown in Figure 12. The F1-scores might improve by the addition of new data to the data-sets since, in general, in deep learning, the more data, the better the final performance is.

## 5. Conclusions

This paper presented the design of a system for the classification of locomotion modes, transitions, and gait phases for both healthy and osseointegrated lower-limb amputee subjects by using IMUs. Different deep neural network configurations are investigated by combining convolutional and recurrent layers. As input to the networks, the frequency-domain information in the form of a spectrogram of one IMU (located on the upper leg) or two IMUs (located on both the upper and lower leg) are used.

The results showed that a system composed of CNN-LSTM networks is able to correctly classify with a mean F1-score of 0.89 ± 0.01 and 0.91 ± 0.01 for the healthy subjects (considering the locomotion modes, transitions, and gait phases), and 0.92 ± 0.01 and 0.95 ± 0.01 for the amputee subject (only locomotion modes and transitions) by using one and two IMUs, respectively. Moreover, it was shown that the generalization capabilities for this type of classification task might be difficult to achieve given the nature of the data that are used, and that healthy and amputee data should not be mixed since they worsen the performance of the classification.

## Figures and Tables

**Table 1:** State of the art of machine learning and deep learning techniques for the classification and/or prediction of (loco-)motion modes, transitions, and gait phases by means of IMU data of healthy and/or impaired subjects. (CNN: convolutional neural network; RNN: recurrent neural network; LSTM: long short-term memory; SVM: support vector machine; QDA: quadratic discriminant analysis; HMM: hidden Markov models; GRU: gated recurrent unit; LDA: linear discriminant analysis; DT: decision trees; KNN: k-nearest neighbors; NB: naive Bayes).

Ref. Year	Method	Features	Accuracy/ Error	IMU Placement	Classes	Subject(s)
**Locomotion Modes and/or Transitions**						
[2] 2020	CNN classification	1 IMU (time domain)	86.7–96.7%	Lower Leg	5 Locomot.	30 Healthy
[3] 2018	RNN (LSTM) classification	2 IMU (time domain)	96%	Upper Arm	5 Locomot.	11 Healthy
[4] 2020	SVM (multi-level) classif., predict.	3 IMU (time domain)	96%, 93%	Upper Leg Lower Leg	5 Locomot. 9 Transit.	10 Healthy
[5] 2020	CNN classif., predict.	3 IMU, 2 EMG, 3 GONIO (freq. domain)	1.1% (error rate)	Upper Leg Lower Leg	5 Locomot. 8 Transit.	10 Healthy
[6] 2019	CNN classif., predict.	3 IMU (time domain)	94.15% 89.23%	Upper Leg Lower Leg	5 Locomot. 8 Transit.	10 Healthy 1 Transf.
[7] 2017	SVM, QDA classif., predict.	2 IMU, load cell time domain	95.8%, 94.9%	Lower Leg	5 Locomot. 8 Transit.	6 Transf.
[8] 2019	HMM classification	1 IMU, 2 pres- sure sensors (time domain)	95.8%	Lower Leg	5 Locomot.	3 Healthy 2 Transf.
[9] 2020	Wavenet classification	2 IMU (time domain)	97.88% (F1-score)	Upper Leg Lower Leg	7 Locomot.	10 Healthy
[10] 2021	RNN (GRU) prediction	2 IMU (time and freq. domain)	93.06% (F1-score)	Upper Leg Lower Leg	8 Locomot. 24 Transit.	1 Transf.
[11] 2021	LDA, DT classification	1 IMU, force sensor (time domain)	2.8% (error)	Lower Leg	5 Locomot.	5 Transt.
[12] 2021	KNN, LD, SVM, NB classification	1 IMU, force sensor (time domain)	0.56% (error rate)	Upper Leg	5 Locomot.	10 Healthy
[13] 2021	CNN classification	Several IMUs (time domain)	98% (F1-score)	Several	9 Locomot.	29 Healthy
[14] 2021	CNN-GRU classification	1 IMU (time domain)	96.54%	Pelvis	18 Activities	51 Healthy
[15] 2022	CNN-LSTM classification	Motion tracker (time domain)	90.89%	Several	12 Activities	20 Healthy
[16] 2020	Geometry-based classif., predict.	1 IMU (time domain)	98.5%	Lower Leg	12 Transit.	3 Healthy 3 Transf.
**Locomotion Modes and/or Gait Phases**						
[17] 2018	LDA, QDA classification	1 IMU (time domain)	100%	Lower Leg	Gait Phases	10 Healthy 5 Transf.
[18] 2016	Thresholding classification	1 IMU (time domain)	99.78%	Lower Leg	Gait Phases	4 Healthy 1 Transf.
[19] 2016	CNN classification	7 IMU (time domain)	97%	Lower Leg	6 Gait Phases	12 Healthy
[20] 2018	QDA (multi-level) classification	2 IMU (time domain)	97%	Upper Leg Lower Leg	Gait Phases	3 Stroke survisors
[21] 2018	Bayesan classification	3 IMU (time domain)	99.87% 99.20%	Lower Leg	3 Locomot. Swing/Stance	8 Healthy
**Locomotion Modes, Transition, and Gait Phases**						
[22] 2018	QDA classification	2 IMU, load cell (time domain)	93.21%	Lower Leg	6 Locomot. 10 Transit. Swing/Stance	3 Transt.
[23] 2014	LDA classif., predict.	3 IMU, pressure insole (time domain)	99.71%	Upper Leg Lower Leg	6 Locomot. 10 Transit. Swing/Stance	7 Healthy

**Table 2:** Number of sequences for locomotion modes and transitions in the ENABL3S data-set.

Label	N. of Sequences	Label	N. of Sequences
S	6639	SA-W	257
W	10009	W-RD	272
RA	3284	RD-W	124
RD	3987	W-ST	531
SA	1304	ST-S	533
SD	1307	W-RA	215
ST	4424	RA-W	262
S-ST	525	W-SD	258
ST-W	128	SD-W	236
W-SA	262		

**Table 3:** Number of sequences for the gait phases in the ENABL3S data-set. W1 corresponds to the gait phase 1 of walking, etc. (see Figure 1).

Label	N. of Sequences	Label	N. of Sequences
W1	937	RD3	682
W2	1813	RD4	981
W3	2049	RD5	1042
W4	1365	SA1	150
W5	1531	SA2	336
W6	1724	SA3	547
W7	2128	SA4	285
RA1	361	SA5	243
RA2	718	SD1	141
RA3	932	SD2	364
RA4	677	SD3	203
RA5	858	SD4	325
RD1	357	SD5	510
RD2	1049		

**Table 4:** Number of sequences for locomotion modes and transitions in the MyLeg data-set.

Label	N. of Sequences	Label	N. of Sequences
S	5963	ST-RA	200
ST	4978	ST-RD	179
W	4182	W-SA	537
SA	2736	W-RA	186
SD	2225	W-S	291
RA	1612	W-SD	193
RD	2208	W-ST	147
S-W	136	SA-ST	71
S-ST	536	SA-W	521
ST-SD	474	SD-ST	77
ST-SA	71	SD-W	513
ST-S	45	RA-RD	427
ST-W	3164	RD-W	483

**Table 5:** Grid search results. Best found hyperparameter value combination for each neural network.

Classif.	Activ.	Learn. Rate	Optimiser	Hidden Units	Dropout	Accuracy
Locom. modes	relu	0.001	RMSProp	[32, 64, 60, 30, 15]	0.25	0.96 ± 0.05
S trans.	relu	0.001	Adam	[32, 64, 60, 30, 15]	0.25	0.96 ± 0.00
W trans.	relu	0.001	Adam	[32, 64, 60, 30, 15]	0.5	0.97 ± 0.00
SA trans.	elu	0.001	RMSProp	[64, 128, 120, 60, 30]	0.25	0.93 ± 0.01
SD trans.	elu	0.001	RMSProp	[64, 128, 120, 60, 30]	0.25	0.92 ± 0.02
RA trans.	elu	0.001	RMSProp	[64, 128, 120, 60, 30]	0.25	0.96 ± 0.01
RD trans.	elu	0.001	RMSProp	[32, 64, 60, 30, 15]	0.25	0.92 ± 0.01
St trans.	relu	0.001	Adam	[32, 64, 60, 30, 15]	0.25	0.96 ± 0.00
W gait ph.	tanh	0.0001	Adam	[64, 128, 120, 60, 30]	0.5	0.92 ± 0.00
SA gait ph.	relu	0.001	RMSProp	[64, 128, 120, 60, 30]	0.25	0.94 ± 0.01
SD gait ph.	tanh	0.001	RMSProp	[32, 64, 60, 30, 15]	0.25	0.92 ± 0.02
RA gait ph.	elu	0.001	Adam	[32, 64, 60, 30, 15]	0.5	0.96 ± 0.01
RD gait ph.	tanh	0.001	Adam	[64, 128, 120, 60, 30]	0.25	0.95 ± 0.00

**Table 6:** F1-scores obtained with the CNN-LSTM with one ENABL3S subject. The row ‘Before’ shows results obtained without the independent subject data during training, while the row ‘After’ shows results obtained with the independent subject data during training. The row ‘ENABL3S’ shows results on ENABL3S data after retraining with the MyLeg data.

	F1-Score 1 IMU	F1-Score 2 IMUs
Before	0.24 ± 0.06	0.21 ± 0.04
After	0.87 ± 0.01	0.95 ± 0.01
ENABL3S	0.33 ± 0.02	0.37 ± 0.03

**Table 7:** F1-scores with CNN-LSTM with 10 ENABL3S subjects. The row ‘Before’ represents results obtained without the independent subject data during training, while the row ‘After’ represents results obtained with the independent subject data during training. The row ‘ENABL3S’ shows results on ENABL3S data after retraining with MyLeg data.

	F1-Score 1 IMU	F1-Score 2 IMUs
Before	0.29 ± 0.01	0.22 ± 0.02
After	0.84 ± 0.02	0.946 ± 0.01
ENABL3S	0.24 ± 0.03	0.34 ± 0.01

**Table 8:** Running time for the classification of one sequence (averaged over 1000 data) when using one and two IMUs.

	Time 1 IMU [ms]	Time 2 IMUs [ms]
Spectrogram	2.45 ± 0.02	4.79 ± 0.04
Classification	36.73 ± 5.61	37.09 ± 6.45
Total	39.19 ± 5.63	41.88 ± 6.49

## Data Availability

Not applicable.
